# Fat-cartilage axis: the regulation of IL-6/Osteopontin signaling in osteoarthritis of mice

**DOI:** 10.1038/s41420-025-02622-6

**Published:** 2025-07-15

**Authors:** Bing-yang Dai, Zhong-lian Huang, Ming-gui Bao, Hong-jiang Chen, Xiao-hui Lu, Jun Hu

**Affiliations:** 1 Department of Orthopaedics, The First Affiliated Hospital of Shantou University Medical College, Shantou, Guangdong China; 2 Orthopaedic Medical Research Center, The First Affiliated Hospital of Shantou University Medical College, Shantou, Guangdong China

**Keywords:** Acute inflammation, Trauma

## Abstract

The infrapatellar fat pad (IPFP) acts as a bioactive reservoir, secreting proinflammatory cytokines that orchestrate both local and systemic inflammatory cascades. Despite its emerging role in knee osteoarthritis (OA) pathophysiology, the molecular and cellular mechanisms driving IPFP-mediated disease progression remain a critical gap in mechanistic understanding. 12-week-old male *C57BL/6* mice underwent either destabilization of the medial meniscus (DMM) surgery or Sham surgery. Here, we find that the extreme sensitivity of IPFP makes it prone to act as a reservoir of inflammatory factors, which may indiscriminately disrupt the stability of its surrounding tissues. We further ascertain the role of IL-6 in initializing fibrosis in IPFP at early stage of OA and modulating osteopontin (OPN) secretion that cascades cartilage deterioration. Notably, removal of the IPFP in DMM mice reverses the abnormal functions of the knee joint. Compromising the progress of fibrosis by intra-IPFP injection of siRNA *Cd61* or inhibition of OPN expression can drastically ameliorate cartilage deterioration. Our findings elucidate a pivotal role for IL-6 in instigating fibrotic remodeling within the IPFP during early-stage OA, concurrently regulating OPN secretion to propagate cartilage matrix degradation. This study thus establishes a conceptual framework for therapeutic intervention by targeting the IL-6/OPN signaling axis in the IPFP during OA initiation, offering a promising strategy to disrupt disease progression.

## Introduction

Knee osteoarthritis (OA) is a chronic degenerative condition characterized by progressive locomotor dysfunction. Current therapeutic strategies for OA remain palliative, with no disease-modifying drugs clinically approved to date, thereby highlighting its substantial socioeconomic burden [1]. The hallmark pathological features of OA encompass progressive degradation of the cartilage extracellular matrix, synovitis, osteophyte formation, and subchondral bone remodeling [2].

Emerging evidence indicates that systemic and intra-articular metabolic factors exhibit a positive correlation with structural joint deterioration in patients with OA [3–5]. However, synovial fluid cytokine profiles diverge markedly from their circulating counterparts in serum, underscoring compartment-specific pathophysiological dynamics [3, 4, 6]. These findings suggest that synovium-dependent secretion disrupts the balance between serum and synovial fluid. Consequently, there is a major focus on synovium-derived cytokines present in synovial fluid. Macroscopically, the infrapatellar fat pad (IPFP) is situated posterior to the patella and adjacent to the synovium [7]. Established theories suggest that the IPFP houses an intricate vascular network fused with synovium, thereby playing a role in the maintenance of cytokine levels [8]. Infiltrating cells, such as monocytes, macrophages, and myofibroblasts, can rapidly respond to environmental fluctuations by secreting various factors under certain conditions [9]. Thus, the IPFP is recognized as an endocrine organ that modulates activities of surrounding tissues through both autocrine and paracrine mechanisms [10].

In early-stage OA mice, proinflammatory cytokines including osteopontin (OPN) and interleukin (IL)-6 are upregulated within the IPFP [10]. Obesity further amplifies IL-6-mediated signaling to drive macrophage infiltration into adipose depots. Conversely, genetic or pharmacological inhibition of this pathway attenuates adipose tissue inflammation and fibrotic remodeling [11, 12]. Recently, IPFP-derived OPN has been regarded as a determinant in OA progression, suggesting that reducing OPN expression may be an effective strategy for mitigating the advancement of OA [10]. Tumor cells exhibit decreased expression of OPN when treated with anti-IL-6 antibodies, indicating that IL-6 serves as an upstream regulator of OPN expression [13, 14]. The scalability of IL-6/OPN axis may also apply to IPFP in addition to tumor. In addition, macrophages may serve as a source of IL-6 secretion, which triggers IL-6 signaling and subsequently upregulates OPN expression [15]. Given the dual roles of IL-6 signaling in both pro-inflammation and anti-inflammation processes, it is challenging to devise an appropriate intervention for tissue regeneration by regulating IL-6 expression [16, 17]. Therefore, targeting OPN, which is considered a downstream product of this pathway, may present a more promising strategy for ameliorating OA progression.

Nevertheless, the mechanistic contributions of the IPFP-derived IL-6/OPN signaling axis to OA pathogenesis remain incompletely characterized. In this study, we demonstrate that the IPFP exhibits dynamic responsiveness to early OA-associated pathological microenvironmental shifts. These adaptations are hallmarked by dysregulated cytokine release and fibrotic remodeling, which collectively disrupt the homeostatic equilibrium of the IPFP and adjacent articular cartilage, thereby driving pathological crosstalk. We confirm the causative role of IPFP-derived IL-6 in mediating the expression of OPN, which is involved in chondrocyte abnormalities. By reducing fibrosis through intra-IPFP injection of siRNA *Cd61*, Neu Ab, or inhibiting OPN expression, chondrocyte injury during OA can be mitigated. Therefore, this study offers a promising strategy for mitigating OA progression by targeting the IL-6/OPN axis in the IPFP at an early stage.

## Results

### Histological and molecular alterations in articular cartilage of early-stage OA mice

**Fig. 1:**
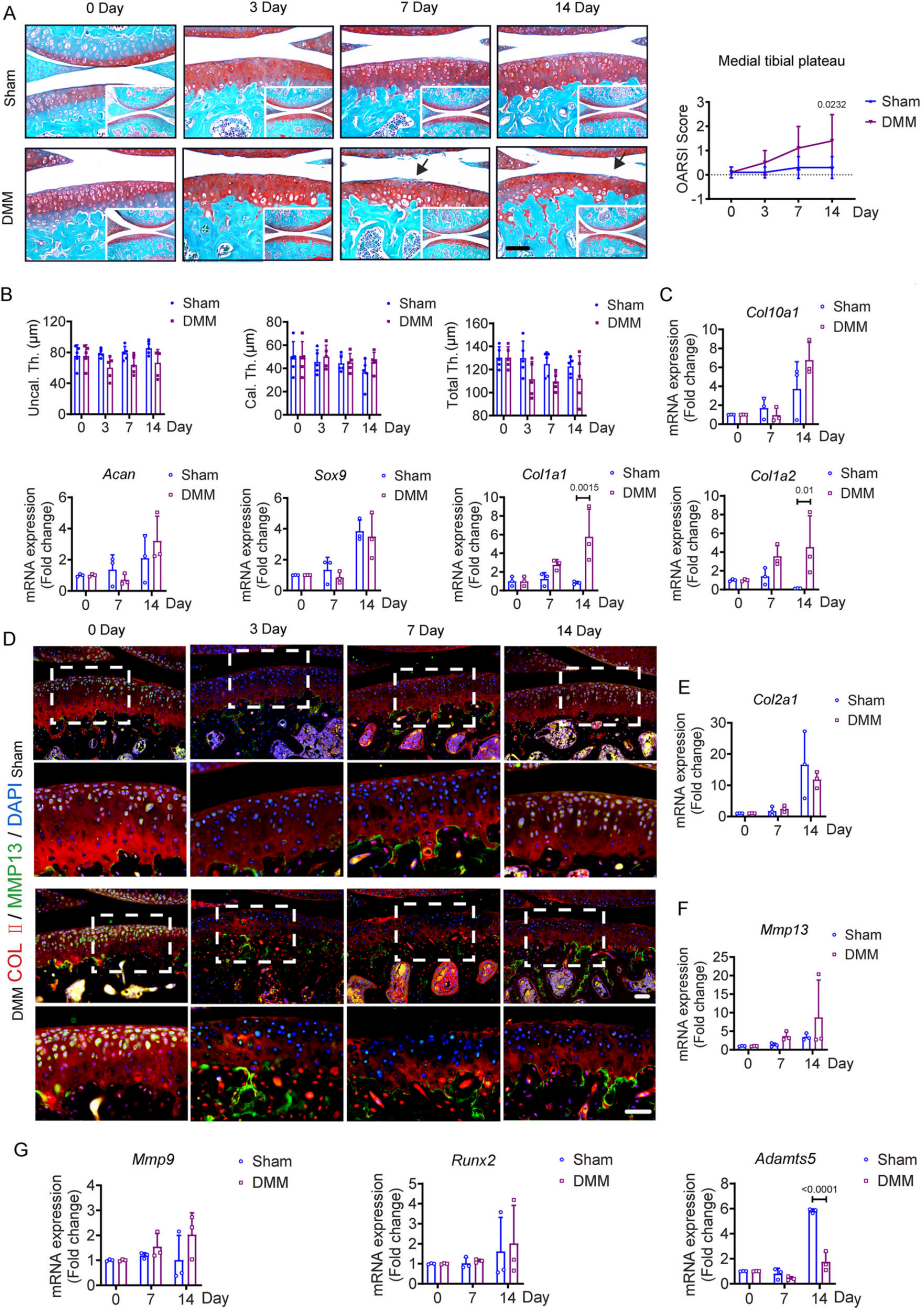
Histological and molecular alterations in articular cartilage of early-stage OA mice. **A** Representative images of Safranin O/Fast Green staining and quantification of OA severity using OARSI scores in knee joint sections from Sham and DMM mice on days 0, 3, 7, and 14. Scale bar: 100 μm. The image is magnified from the lower right corner. *n* = 5 mice per group. **B** Quantification of the average thicknesses of calcified cartilage thickness (Cal. Th.), uncalcified cartilage thickness (Uncal. Th.), and total cartilage thickness (Total Th.) in Sham and DMM mice on days 0, 3, 7, and 14. *n* = 5 mice per group. **C** Relative expressions of *Col10a1*, *Acan*, *Sox9*, *Col1a1*, and *Col1a2* in cartilage from Sham and DMM mice on days 0, 7, and 14. Each group consists of 3 biologically independent samples, with each sample pooled from four mice. **D** Representative images of immunofluorescence staining of COL II (red), MMP13 (green), and nucleus (blue) in articular cartilage from Sham and DMM mice on days 0, 3, 7, and 14. Scale bar: 50 μm. The bottom row shows magnified views from the dotted frame in top row. Scale bar: 50 µm. (**E**, **F**) Relative expressions of *Col2a1* (**E**) and *Mmp13* (**F**) in cartilage from Sham and DMM mice on days 0, 7, and 14. Each group consists of 3 biologically independent samples, with each sample pooled from four mice. **G** Relative expression of *Mmp9*, *Runx2*, and *Adamts5* in cartilage from Sham and DMM mice on days 0, 7, and 14. Each group consists of 3 biologically independent samples, with each sample pooled from four mice. All data are presented as mean ± SD. Two-way ANOVA with *Sidak’s post hoc* test (**A**, **B**, **C**, **E**, **F**, **G**) was used.

The mice underwent either destabilization of the medial meniscus (DMM) surgery or Sham surgery. We found the superficial fibrillation without cartilage matrix loss on day 7 post-DMM surgery and observed the minute vertical-cleft and loss of surface lamina in the articular cartilage of the medial tibial plateau on day 14 post-DMM surgery (Fig. 1A). All the histopathological grades were quantified by Osteoarthritis Research Society International (OARSI). The score was significantly increased in DMM mice relative to the Sham mice on day 14 post surgeries (Fig. 1A). The thickness of uncalcified cartilage (Uncal. Th) or the thickness of calcified cartilage (Cal. Th.) were comparable between both groups on days 3, 7, and 14 post surgeries, reflecting the similar total cartilage thickness (Total Th.) in the articular cartilage before day 14 post surgery (Fig. 1B). Gene expression of chondrocyte-specific mRNA (*Col10a1*, *Acan*, and *Sox9*) with RT-qPCR displayed no significant difference between the two groups in the cartilage on days 0, 7, and 14 post surgeries (Fig. 1C). Hypertrophy markers of cartilage matrix components (*Col1a1* and *Col1a2*) starkly increased in the DMM group compared to that in the Sham group on day 14 post surgeries (Fig. 1C). Co-immunofluorescence staining demonstrated a decreased content of COL2A1, a chondrogenic marker, but a similar expression level of MMP13 in articular cartilage of both groups (Fig. 1D). However, no difference was found in the mRNA expressions of *Col2a1*, *Mmp13*, *Mmp9*, and *Runx2* in the cartilages of both groups (Fig. 1E–G). Furthermore, the RT-qPCR data displayed decline of *a disintegrin and metalloproteinase with thrombospondin motifs 5* (*Adamts5*), a primary enzyme responsible for the degradation of aggrecan in the cartilage matrix, in the DMM group (Fig. 1G). These molecular results contradicted the morphological findings, suggesting that the external/systemic factors of articular cartilage may underpin the progressive cartilage damage.

### Fibrotic alteration in the IPFP of early-stage OA mice

**Fig. 2:**
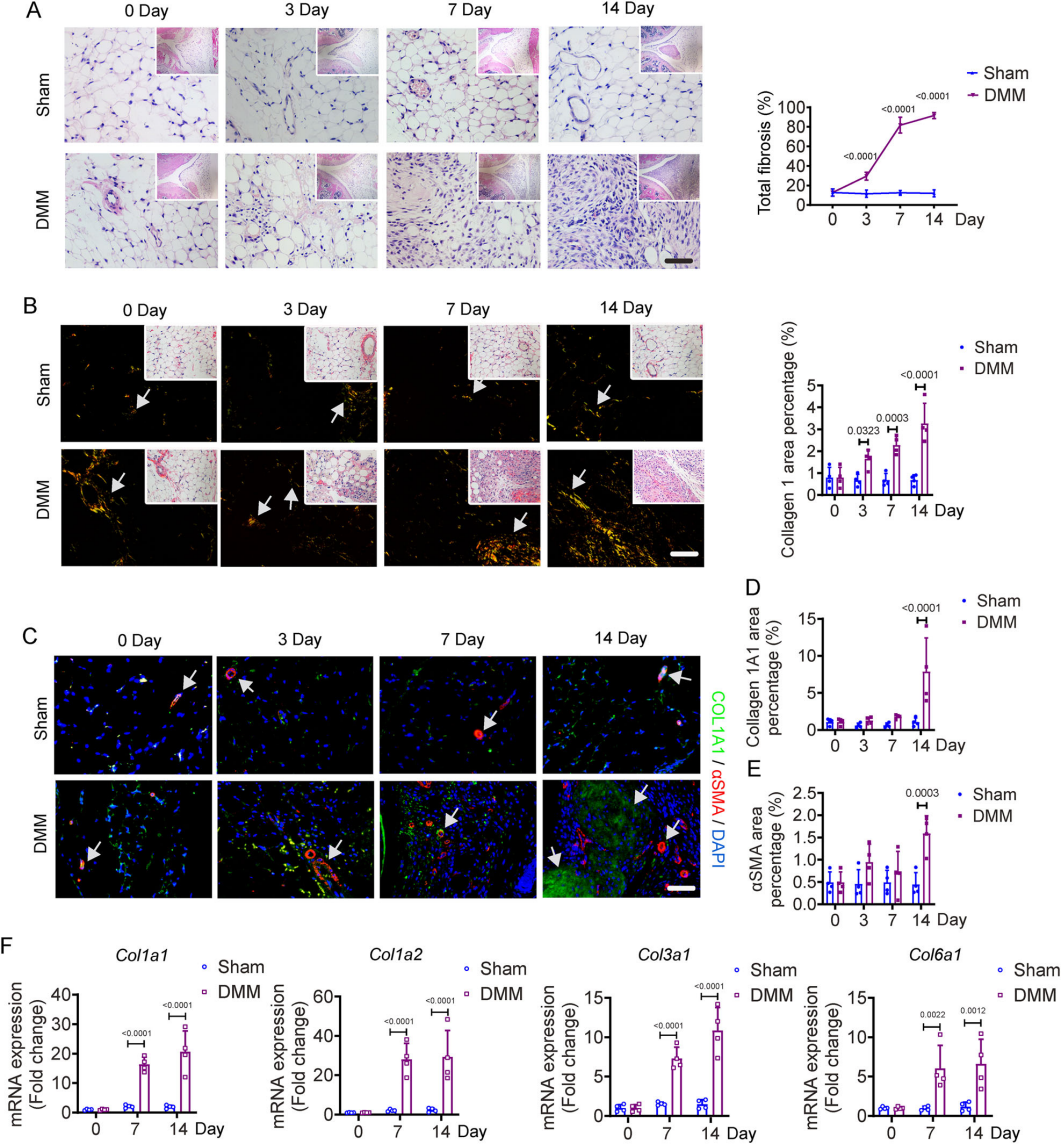
Fibrotic alteration in the IPFP of early-stage OA mice. **A** Representative image of H&E staining and quantification of IPFP from Sham and DMM mice on days 0, 3, 7, and 14. *n* = 5 mice per group. Scale bar: 50 µm. The image is magnified from the top right corner. **B** Representative image of Picro-Sirius Red staining and quantification of the Col I area percentage in the IPFP from Sham and DMM mice on days 0, 3, 7, and 14. *n* = 4 mice per group. Scale bar: 50 μm. **C-E** Representative images (**C**) and quantifications (**D**, **E**) of immunofluorescence staining of COL1A1(green), αSMA (red), and nucleus (blue) in IPFP from Sham and DMM mice on days 0, 3, 7, and 14. *n* = 4 mice per group. Scale bar: 50 μm. **F** Relative expression of *Col1a1*, *Col1a2*, *Col3a1*, and *Col6a1* in IPFP from Sham and DMM mice on days 0, 7, and 14. Each group consists of 4 biologically independent samples, with each sample pooled from six mice. All data are presented as mean ± SD. Two-way ANOVA with *Sidak’s post hoc* test (**A**, **B**, **D**, **E**, **F**) was used.

The IPFP, nearby the anterior medial meniscus, showed mild fibrosis on day 3 post-DMM surgery, which progressed over time, with no similar fibrotic changes in Sham mice (Fig. 2A). The representative images and fibrosis quantification on day 14 revealed a substantial replacement of adipocytes with a dense connective tissue (Fig. 2A). Polarized light observation of Picro-Sirius Red stained sections showed that the significant densification in IPFP was due to substantial collagen I synthesis, which was also validated by immunofluorescence staining of COL1A1 (Fig. 2B–D). Immunofluorescence analysis of alpha-smooth muscle actin (αSMA), a representative marker of myofibroblast, displayed an increased amount of vascular lumen-like structures in DMM mice relative to the Sham mice (Fig. 2C, E). All fibrosis-related pathogenic markers (*Col1a1*, *Col1a2*, *Col3a1*, and *Col6a1*) were significantly elevated in the IPFP of DMM mice compared to Sham mice (Fig. 2F). These findings suggest that the fibrotic changes in the IPFP occur earlier than those in the articular cartilage of OA mice.

### The inflammatory responses of IPFP at early-stage OA mice

**Fig. 3:**
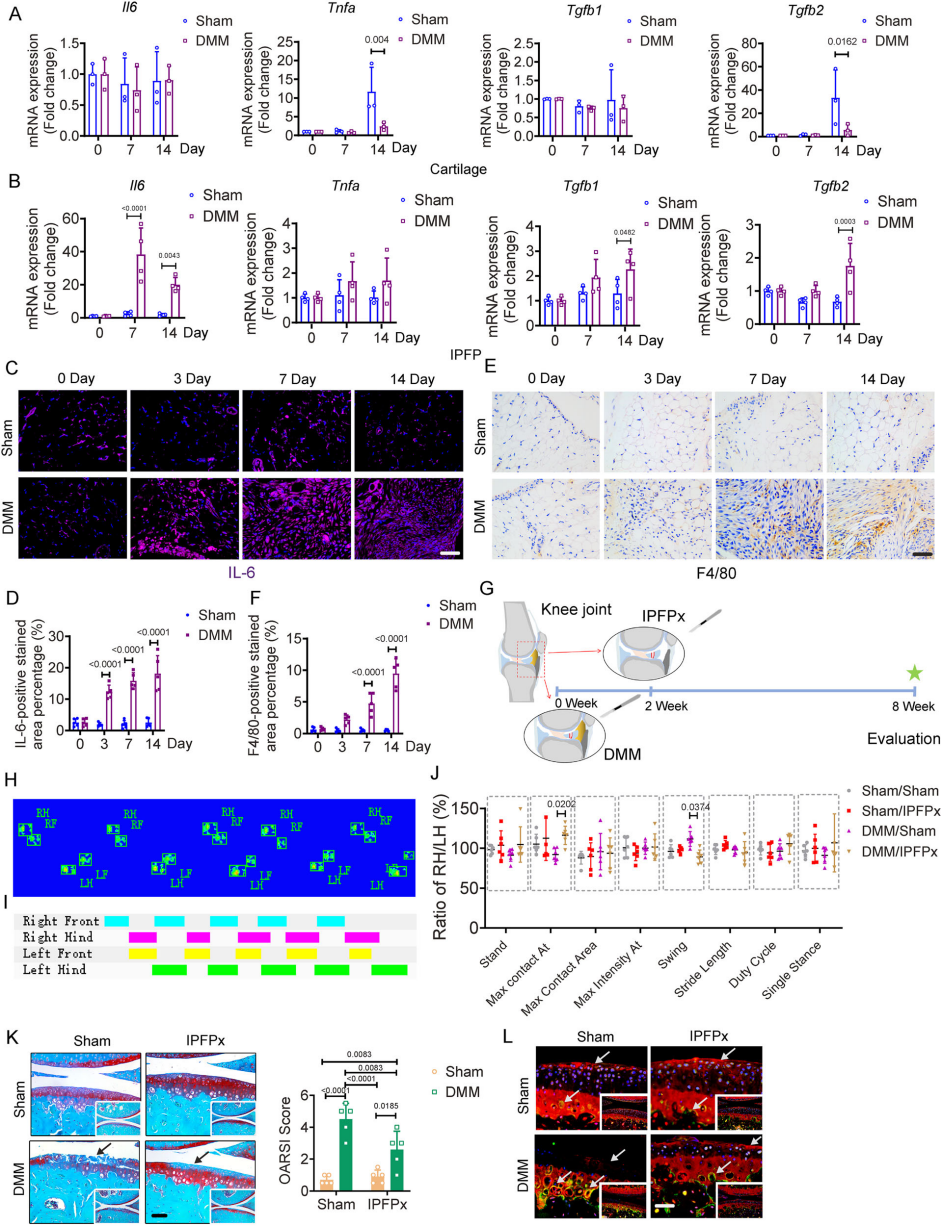
The role of IPFP in OA progression. **A** Relative expression of *Il-6*, *Tnfa*, *Tgfb1*, and *Tgfb2* in cartilage from Sham and DMM mice on days 0, 7, and 14. Each group consists of 3 biologically independent samples, with each sample pooled from four mice. **B** Relative expression of *Il-6*, *Tnfa*, *Tgfb1*, and *Tgfb2* in IPFP from Sham and DMM mice on days 0, 7, and 14. Each group consists of 4 biologically independent samples, with each sample pooled from six mice. **C and D** Representative images (**C**) and quantifications (**D**) of immunofluorescence staining for IL-6 (purple) and nucleus (blue) in IPFP from Sham and DMM mice on days 0, 3, 7, and 14. *n* = 5 mice per group. Scale bar: 50 μm. **E and F** Representative images (**E**) and quantifications (**F**) of immunohistochemical staining for F4/80 in IPFP from Sham and DMM mice on days 0, 3, 7, and 14. *n* = 5 mice per group. Scale bar: 50 μm. **G** Schematic diagram illustrating the experimental outline. Surgical removal of the inflamed IPFP on day14 post-Sham or -DMM surgery. **H**–**J** Images display pawprints of the right forelimb (RF), right hind limb (RH), left forelimb (LF), and left hind limb (LH) of a running mouse, captured by automated gait analysis (Catwalk). Representative catwalk gait analysis among groups on day 56 post-Sham or -DMM surgery. *n* = 6 mice per group. **K** Representative Safranin O/Fast Green staining and quantification of OA severity using OARSI scores among groups on day 56 post-Sham or -DMM surgery. *n* = 5 mice per group. Scale bar: 100 µm. **L** Representative images of immunofluorescence staining for COL II (red), MMP13 (green), and nucleus (blue) in articular cartilage on day 56 post-Sham or -DMM surgery. Scale bar: 50 μm. The image is magnified from the lower right corner. All data are presented as mean ± SD. Two-way ANOVA with *Sidak’s post hoc* test (**A**, **B**, **D**, **F**) and Two-way ANOVA with *Tukey’s post hoc* test (**J**, **K**) were used.

Inflammation may act as a critical factor in joint destruction by promoting articular cartilage degradation [18]. The inflammatory markers (*Il6*, *Tnfa*, *Tgfb1*, and *Tgfb2*) are typical of OA disease involving in cartilage degradation, with IL-6 being particularly responsible for stimulating protein synthesis at early stage [18, 19]. The expression levels of the aforementioned markers were comparable between two groups on days 0, 7, and 14 post surgeries, except for the lower expressions of *Tnfa* and *Tgfb2* in articular cartilage of DMM mice compared to Sham mice on day 14 post surgery (Fig. 3A). The early presence of fibrosis in the IPFP suggests the possibility of a substantial set of secretory cytokines originating from the IPFP. We found that the gene expression of *Il6* was significantly greater in the IPFP of the DMM group than in the Sham group on both days 7 and 14 post surgery (Fig. 3B), corresponding to the results displayed in immunofluorescence staining of IL-6 (Fig. 3C, D). The higher expression levels of *Tgfb1* and *Tgfb2* may be induced by the increased expression of *Il6* in DMM group after day 7 post surgery (Fig. 3B). Macrophage can secrete a wide range of inflammatory cytokines that regulate inflammation, including IL-6 [20]. In addition to IL-6 expression, abnormal presence of macrophages was observed in IPFP of DMM mice on both days 7 and 14 post DMM surgeries (Fig. 3E, F).

Due to the extensive fibrotic alterations and inflammatory responses in IPFP occurred on day 14 in DMM mice, removal of inflamed IPFP was proposed to arrest the OA progression driven by cytokines. We removed the IPFP (IPFPx) on day 14 post-Sham or -DMM surgery prior to the articular cartilage degradation (Fig. 3G). The acquisition of duty cycle, maximal contact At, stand, maximal intensity At, maximal contact area, swing, single stance, and stride length were calculated for profiling an obscure difference in locomotion on day 56 post surgery. These results were used to detect the mechanical instability caused by surgical treatments or pain [10]. The removal of the IPFP in DMM mice reversed the abnormal functions quantified by the parameters, such as maximal contact At and swing (Fig. 3H–J). Representative images of Safranin O/Fast Green staining, quantified by OARSI score, showed reduced articular cartilage destruction in the IPFPx/DMM group compared to the Sham/DMM group on day 56 post-Sham or -DMM surgery (Fig. 3K). Co-immunofluorescence staining displayed the greater content of COL2A1 and a smaller expression of MMP13 in articular cartilage of IPFPx/DMM group than that in the Sham/DMM group on day 56 (Fig. 3L). These results indicate that cytokines derived from the IPFP at the early stage can exacerbate the progression of OA.

### IPFP-derived IL-6 triggers OA progression

**Fig. 4:**
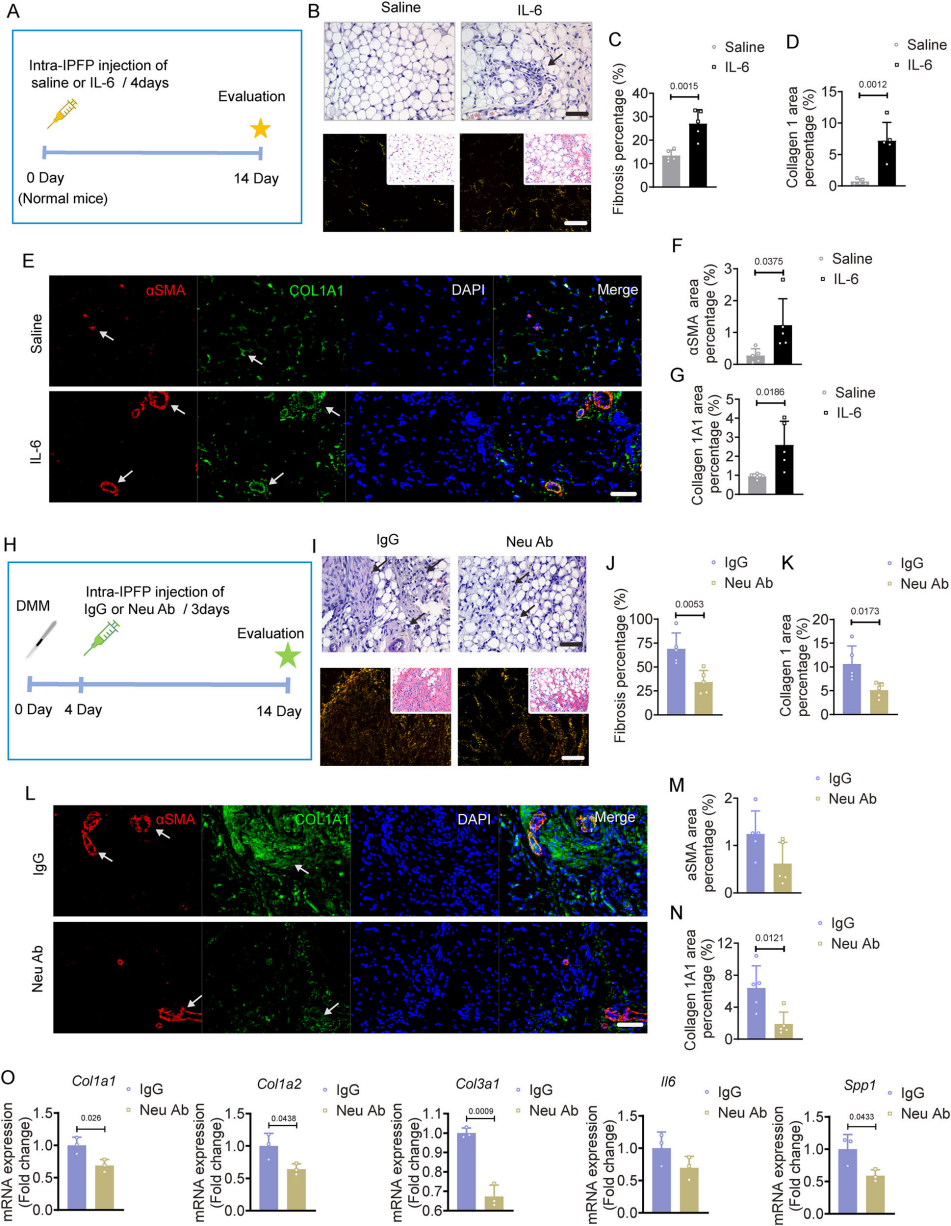
The role of IPFP-derived IL-6 in OA progression. **A** Schematic diagram illustrating experimental design. Recombinant IL-6 protein (1 μg/unilateral/4 days) or saline (15 μL/unilateral/4 days) was locally injected into the subcapsular IPFP without any surgery. All mice were sacrificed on day 14. **B** Representative images of H&E (top row) and Picro-Sirius Red staining (bottom row) of IPFP from saline- and IL-6-treated mice on day 14 post injections. Scale bar: 50 μm. **C**, **D** Matched quantifications of fibrosis area and collagen I area from (**B**). *n* = 5 mice per group. **E-G** Representative images (**E**) and quantifications (**F**, **G**) of immunofluorescence staining for COL1A1(green), αSMA (red), and nucleus (blue) in IPFP on day 14 post injections. *n* = 5 mice per group. Scale bar: 50 μm. **H** Schematic diagram illustrating the experimental outline. An IL-6 neutralizing antibody (Neu Ab) (2 μg/unilateral/3 days) or IgG (15 μL/unilateral/3 days) was locally injected into the subcapsular IPFP on day 4 post-Sham and -DMM surgeries. All mice were sacrificed on day 14 post-Sham and -DMM surgeries. **I** Representative images of H&E (top row) and Picro-Sirius Red staining (bottom row) of IPFP from IgG and Neu Ab-treated mice on day 14 post-Sham and -DMM surgeries. Scale bar: 50 μm. **J**, **K** Corresponding quantifications of fibrosis area and collagen I area from (**I**). *n* = 5 mice per group. **L-N** Representative images (**L**) and quantifications (**M**, **N**) of immunofluorescence staining for COL1A1(green), αSMA (red), and nucleus (blue) in IPFP on day 14 post surgeries. *n* = 5 mice per group. Scale bar: 50 μm. **O** Relative expressions of *Col1a1*, *Col1a2*, *Col3a1*, *Il-6*, and *Spp1* in IPFP on day 14 post surgeries. Each group consists of 3 biologically independent samples, with each sample pooled from four mice. All data are presented as mean ± SD. Two-tailed *t*-test (**C**, **D**, **F**, **G**, **J**, **K**, **M**, **N**, **O**) was used.

IL-6 is unambiguously demonstrated as a vital stimulator for the proteins’ synthesis in the early stage [21]. Then to examine whether IL-6 is involved in the fibrosis of IPFP at early-stage of OA progression, we locally injected recombinant IL-6 into the subcapsular IPFP of normal mice without making a surgical incision (Fig. 4A). The control mouse received the same volume of saline as a control (Fig. 4A). Fibrosis was significantly greater in the IPFP of mice treated with IL-6 compared to the saline-injected group (Fig. 4B, C). Polarized light observation of the IPFP showed increased densification in IPFP due to substantial collagen I synthesis (Fig. 4B, D). Co-immunofluorescence staining of αSMA and COL1A1 displayed increased fibrosis and vascularization in IPFP of mice treated with IL-6 compared to those treated with saline (Figs. 4E–G). Conversely, we investigated whether depleting IL-6 at the early stage could reduce IPFP fibrosis during OA progression. Then we locally injected IL-6-neutralizing antibody (Neu Ab) or matched IgG into the subcapsular IPFP in DMM mice on day 4 post surgery (Fig. 4H). Neu Ab treatment significantly reduced fibrosis progression in the IPFP compared to the IgG-treated group (Fig. 4I, J). Picro-Sirius Red staining with polarized light analysis validated the decreased expression of collogen I in IPFP treated with Neu Ab (Fig. 4I, K). Co-immunofluorescence staining of αSMA and COL1A1 showed reduced fibrosis and vascularization in IPFP of mice treated with Neu Ab compared to those receiving IgG treatment (Fig. 4L–N). Additionally, Neu Ab intervention in the IPFP significantly suppressed the expression of fibrosis-related proteins, including *secreted phosphoprotein 1* (*Spp1*), *Il6*, *Col1a1*, *Col1a2*, and *Col3a1* (Fig. 4O).

Altogether, these results suggest that IPFP-derived IL-6 plays a causative role in the inflammatory responses of knee joint, especially in the vascularization and fibrosis of IPFP in OA mice.

### IPFP-derived IL-6 induces the OPN expression involved in the progression of articular cartilage destruction

**Fig. 5:**
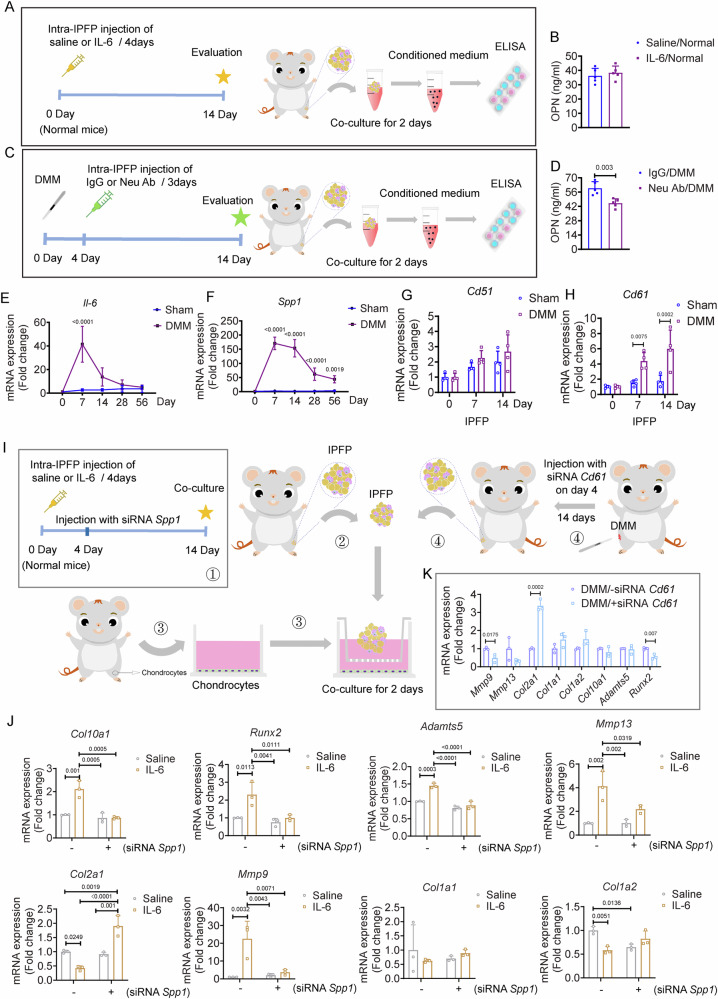
The role of IL-6/OPN axis in IPFP during OA progression. **A** Schematic diagram illustrating experimental design. Recombinant IL-6 protein (1 μg/unilateral/4 days) or saline (15 μL/unilateral/4 days) was locally injected into the subcapsular IPFP without any surgical intervention. All mice were sacrificed on day 14 to collect IPFP samples. The concentration of IPFP-derived Osteopontin (OPN) in the conditioned medium was measured by ELISA. **B** Concentrations of IPFP-derived OPN in conditioned medium from (A). *n* = 5 mice per group. **C** Schematic diagram illustrating the experimental outline. Neu Ab (2 μg/unilateral/3 days) or IgG (15 μL/unilateral/3 days) was locally injected into subcapsular IPFP on day 4 post-Sham and -DMM surgeries. All mice were sacrificed on day 14 post-Sham and -DMM surgeries. **D** Concentrations of IPFP-derived OPN in conditioned medium from (**C**). *n* = 5 mice per group. Relative expressions of *Il-6* (**E**) and *Spp1* (**F**) in IPFP from Sham and DMM mice on days 0, 7, 14, 28, and 56 post surgeries. Each group consists of 4 biologically independent samples, with each sample pooled from six mice. Relative expressions of *Cd51* (**G**) and *Cd61* (**H**) in IPFP from Sham and DMM mice on days 0, 7, and 14 post surgeries. Each group consists of 4 biologically independent samples, with each sample pooled from six mice. **I** Schematic diagram illustrating the experimental outline. Step 1: Normal mice received intra-IPFP injections of saline or IL-6 every 4 days and were simultaneously administered siRNA *Spp1* or not on day 4; Steps 2 and 3: IPFP collected on day 14 from step 1 was co-cultured with primary chondrocytes collected from young mice; Step 4: DMM mice received intra-IPFP injections of siRNA *Cd61* or not on day 4 post DMM surgery; Steps 3 and 4: IPFP collected on day 14 from step 4 was co-cultured with primary chondrocytes collected from young mice. **J** Relative expression of *Clo10a1*, *Runx2*, *Adamts5*, *Mmp13*, *Clo2a1*, *Mmp9*, *Col1a1*, and *Col1a2* in primary chondrocytes from steps 2 and 3 in (**I**). *n* = 3. **K** Relative expression of *Mmp9*, *Mmp13*, *Clo2a1*, *Col1a1*, *Col1a2*, *Clo10a1*, *Adamts5*, and *Runx2* in primary chondrocytes from steps 3 and 4 in (**I**). *n* = 3. All data are presented as mean ± SD. Two-tailed *t*-test (**B**, **D**, **K**), Two-way ANOVA with *Sidak’s post hoc* test (**E**, **F**, **G**, **H**), and Two-way ANOVA with *Tukey’s post hoc* test (**J**) were used.

In response to inflammation, synovial cells and macrophages in IPFP may produce OPN that promotes cell migration, cell adhesion, and extracellular matrix fibrosis [10, 22]. We hypothesized that IPFP-derived IL-6 was involved in OPN production at early stage of OA. ELISA results revealed no significant difference in OPN secretion levels between the saline- and IL-6-injected groups within the subcapsular IPFP of normal mice (Fig. 5A, B). However, the level of IPFP-derived OPN in the conditioned medium was significantly reduced when the IPFP from DMM mice was injected with Neu Ab into the subcapsular IPFP (Fig. 5C, D). We found that the *Il6* expression in IPFP peaked on day 7 and regressed until day 56 post DMM surgery (Fig. 5E). The mRNA expression of *Spp1* in IPFP was significantly and persistently higher in DMM mice compared to the Sham mice (Fig. 5F). There was comparable expression of *Cd51* in IPFP of both groups across time points, but significantly increased expression of *Cd61* in IPFP of the DMM group on day 14 post surgery (Fig. 5G, H).

We further characterized the role of the IL-6/OPN axis in IPFP in articular cartilage damage at early stage of OA. First, mice without surgery were intra-IPFP injected with saline or IL-6 and simultaneously received siRNA *Spp1* or not on day 4 as step 1 shown in Fig. 5I. We ex vivo co-cultured the IPFP from step 1 with primary chondrocytes for 2 days (Fig. 5I, routines 1, 2, and 3). IL-6 pre-treated IPFP induced higher expressions of *Clo10a1*, *Runx2*, *Adamts5*, *Mmp13*, and *Mmp9* in primary chondrocytes relative to the control group, while siRNA-mediated *Spp1* knockdown arrested their expressions (Fig. 5J). In addition, siRNA *Spp1* treatment rescued *Col2a1* expression in chondrocytes which was inhibited by IL-6 treatment (Fig. 5J). In addition, we injected siRNA *Cd61* into the subcapsular IPFP on day 4 post-DMM surgery shown as step 4 in Fig. 5I. We ex vivo co-cultured the IPFP from step 4 with primary chondrocytes for 2 days (Fig. 5I, routines 3 and 4). siRNA *Cd61* pre-treated IPFP significantly inhibited the expressions of *Mmp9* and *Runx2* and increased *Col2a1* expression relative to the negative siRNA *Cd61* pre-treated group (Fig. 5K). These results suggest that the IL-6/OPN axis in IPFP is involved in articular cartilage degradation at the early stage of OA.

## Discussion

Current research on OA predominantly focuses on cartilage-centric pathologies. The cartilage extracellular matrix (ECM), being avascular and aneural, is composed solely of chondrocytes [23]. However, emerging evidence reveals that matrix degradation byproducts and pro-inflammatory mediators predominantly originate from adjacent synovial and IPFP tissues through paracrine or autocrine signaling mechanisms. These bioactive factors propagate a feedforward loop of chondrocyte dysregulation, disrupting cartilage homeostasis and functional integrity [23]. Our study reveals a previously unidentified IL-6/OPN axis in IPFP that contributes to cartilage damage during the progression of OA. Blocking this axis at an early stage may be an effective strategy for preventing OA.

To avoid damaging the IPFP, all intra-articular procedures for the DMM model were meticulously performed under a stereo zoom microscope. Despite this, we still observed significant densification replacing the original resident adipocytes in the IPFP following DMM surgery. There was a plethora of cells infiltration coupled with fibrosis and angiogenesis within the IPFP at the early stage of OA. Immune cells are key architects in shaping the progression of fibrosis. They possess a dual nature, capable of either ameliorating or aggravating tissue remodeling [24, 25]. Immune cells are dependent on activation of mesenchymal cells to remodel ECM [26]. Inflamed IPFP may initialize cartilage damage through paracrine signaling that primarily functions to reconstruct the IPFP. Macroscopically, we found time lags of histological change and mRNA expression on inflammation between IPFP and cartilage post-DMM surgery. IPFP responded sensitively to injury with higher expression of IL-6. These swift responses in IPFP decided synchronous modulation of adjacent tissues.

Large numbers of nerves cover and penetrate the IPFP, perceiving perturbations [27]. The basal structure of enlarged adipocytes is responsible for the sensitivity of IPFP. Nevertheless, unlike most other tissues, the IPFP fails to induce adequate vascularization due to the hypoxic response of adipose tissue. Instead, hypoxic adipose tissue induces the expression of pro-fibrotic genes, leading to tissue fibrosis [28]. The necrosis of adipocytes leads to infiltration and activation of immune cells for cytokines secretion [28]. In addition, computational trajectory analysis from single-nuclei RNA sequencing of human IPFP revealed IPFP and synovium represent an integrated tissue unit [29]. Synovial inflammation can trigger fibroblast-like synoviocytes to infiltrate in the mesenchyme of IPFP, increasing and perpetuating IPFP damage. Macrophages resident in IPFP release high levels of cytokines as responses to inflammation, including IL-6 to promote fibrosis [10, 29, 30].

Activated macrophages upregulate OPN expression via the IL-6 pathway [15]. Mononuclear phagocytes (monocytes and macrophages) display high plasticity in both type and number after injury and during fibrosis [31]. Human cardiac *Spp1*^*+*^ macrophages exhibit the highest profibrotic potential compared to other mononuclear phagocytes [26]. *Spp1*^*+*^ macrophages engage in the remodeling of ECM via TGFB1 signal in fibroblasts, thereby elevating the expression of MMPs and the deposition of collagens in colorectal cancer [32]. Akin to our findings, *Spp1*^*+*^ macrophages in visceral adipose tissue are responsible for the chronic inflammation in both local and systemic environments, fostering fibrosis in local via autocrine and bone remodeling via remote control [33]. Hence, OPN, especially when derived from macrophages, is crucial for tissue reconstruction, which may contribute to tissue fibrosis.

Fibrosis stops acute injury, but fibrotic tissue loses the functions of the original tissue. If highly progressive, fibrosis will affect adjacent tissue in the body [34]. OPN is essential for TGFB1-induced expression of α-SMA and extradomain-A in differentiation and activation of myofibroblasts [35]. We aim to restore the microstructure of the IPFP due to its role in buffering mechanical stress during knee joint cycles. After validating the roles of IL-6/OPN axis in the pathophysiology of IPFP fibrosis and the progression of OA, targeting the IL-6/OPN axis presents a potential therapeutic strategy to halt these processes.

Joint cartilage compromises ECM and chondrocytes. Chondrocytes interact with ECM via CD44 and integrins including α1β1, α2β1, αvβ3, α6β1, and α5β1 [36]. The interaction between CD44 and MMP9, the latter being a crucial protein involved in ECM degradation, plays a significant role in the invasion of cancer cells [37]. Higher expression of integrin β3 has a strong correlation with the ossification of subchondral bone in OA progression [10]. OPN interacts with integrins and CD44 to regulate a variety of physiological and pathological processes, including differentiation, proliferation, inflammation, and metabolism [38]. Akin to these findings, OPN plays roles in articular metabolism by regulating the subchondral bone matrix and the ECM components of articular cartilage [39–41]. OPN expression is rarely expressed overflowing the tidemark and strongly existed in the deep zone nearby the calcified cartilage [10]. In addition, the abundance of OPN is detected in deep zone chondrocytes and in clusters of proliferating chondrocytes from osteoarthritic samples with severe lesions [42]. These findings suggest the possibility of OPN in regulating destiny of chondrocytes and calcification of ECM. Our investigation validates that intervention in the IL-6/OPN axis offers the possibility to inhibit degenerative advancement in chondrocytes.

Additional investigations are needed for our findings. First, there is no explanation regarding the interaction and differences in inflammation between synovium and IPFP. Second, we have not ruled out the possibility of OPN source from other tissue (such as circulation or synovium) and other cytokines (such as IL-1β and MMPs) from IPFP involving in the progression of OA. Third, we only measure the expressions of integrins αv and β3 as they are the most highly upregulated subunits in OA tissues [43], we still cannot exclude the involvement of other receptors of OPN. Due to modulation of fibrosis is complicated, there is a limited effect of intra-IPFP injecting Nue Ab on attenuating fibrosis. Then, cocktail treatment targeting multiple factors in fibrosis may be more effective.

Our study delineates a pathogenic mechanism wherein the IL-6/OPN signaling axis within the IPFP mediates pathological crosstalk, driving articular cartilage degeneration and fibrotic remodeling of the IPFP. Therapeutic attenuation of IL-6 or OPN via intra-IPFP delivery of neutralizing antibodies or siRNA demonstrates preclinical efficacy in ameliorating chondrocyte dysfunction and fibro-inflammatory pathology, thus positioning targeted modulation of this axis as a tractable strategy for early osteoarthritis intervention. Taken together, this study suggests that IPFP inflammation plays a crucial role in initiating OA and that blocking the IL-6/OPN axis in the IPFP could serve as an innovative therapeutic strategy to prevent OA progression.

## Materials and methods

### Animal

The male *C57BL/6* mice, including both 12-week-old (25–30 g) and 5-week-old mice, were used in this study. Mice were housed at the animal center under a 12-hour light/dark cycle, with an ambient temperature of 18–24 °C, 70% humidity, and provided with food and water *ad libitum*. All surgical procedures were conducted in accordance with standard protocols and were approved by the Animal Experiments Ethics Committee of Shantou University (Approval No. SUMC2022-132). Mice showing signs of suffering during the study were humanely euthanized. Although the treatments for animal were not blinded, the analysis was performed by three independent investigators.

### Mouse knee osteoarthritis model

The mice underwent either Sham surgery or destabilization of the medial meniscus (DMM) surgery on the right hind limb. Details regarding the DMM surgery were briefly described as below [10]. Following anesthesia with ketamine (60 mg/kg, i.p.) and xylazine (4 mg/kg, i.p.), the skin and joint capsule of 12-week-old male *C57BL/6* mice were exposed at the medial parapatellar region. A 25-gauge (G) needle was used to identify and transect the anterior horn of the medial meniscus from the less fatty area of the IPFP. The medial meniscus was then allowed to displace medially. 7-0 prolene sutures and 5-0 vicryl sutures were used to close the incisions in layers in an interrupted pattern. To consider avoiding damages to the blood supply of the IPFP, all intra-articular manipulations were finished using a stereo zoom microscope. For the Sham mouse, the joint capsule of right hind limb was opened without any manipulating the joint tissues. All procedures were performed under aseptic.

### Histological analysis

Samples were fixed in 4% paraformaldehyde (PFA) solution for 24 hours. Then samples were washed with distilled water and decalcified the calcium matrix in 12.5% ethylenediaminetetraacetic acid (EDTA, pH 7.4, Sigma, cat# 798681) for 2 weeks at room temperature. Paraffin-embedded samples were sectioned at 5-7 μm thickness. Section staining with Picro-Sirius Red, Haematoxylin and Eosin (H&E), and Safranin O/Fast Green (Sigma, cat# S8884/F7258) were routinely performed as previously described [10, 44, 45]. To quantify the thickness of articular cartilage, we divided the tibial plateau into quarters from the medial collateral ligament side to the medial intercondylar nodes, corresponding to 25%, 50%, and 75% points. Sections from each sample at these three points were stained with Safranin O/Fast Green and outlined to quantify the thickness using ImageJ (version 1.52v, USA) software [46].

### Immunohistochemical and immunofluorescence staining

Sections were deparaffinized by absolute xylene and rehydrated by gradient alcohols and distilled water. For immunohistochemical staining [47], the solution containing 3% hydrogen peroxide was used to quench the endogenous peroxidase activity for 15 min in the dark. Citrate buffer (10 mM sodium citrate, 0.05% Tween 20, pH 6.0) was used to significantly improve immunohistochemical staining for 30 min at 80 °C. Sections were immersed in blocking buffer (1% bovine serum albumin (BSA, Sigma, USA, cat# A7906) and 0.1% Triton X-100 in phosphate-buffered saline (PBS)) to block the non-specific binding sites for 30 min. Sections were incubated with the primary antibody recognizing F4/80 (Abcam, cat# ab6640, 1:200) overnight at 4 °C. The primary antibody or specified secondary antibody (Abcam, cat# ab6734,1:200) was diluted in BSA buffer (1% BSA in PBS). The secondary antibody was added for 1-2 hours at room temperature. Positive stain was detected by the 3,3′-Diaminobenzidine (DAB, Abcam, cat# AB64238). Negative control was incubated with the secondary antibody without the primary antibody. All sections were stained with hematoxylin for nucleus counterstain.

For immunofluorescence staining [10, 47], rehydrated sections were treated with citrate buffer (at 80 °C) for 30 min, and blocked with blocking buffer for 30 min. The primary antibody recognizing IL-6 (Thermofisher, cat# AMC0862, 1:200), COL1A1 (Abclonal, cat# A16891, 1:50), MMP13 (Abcam, cat# ab39012, 1:200), COL2A1 (Santa, cat# M2139, 1:50), or αSMA (Thermofisher, cat# MA5-11547, 1:200) was diluted in 1% BSA buffer. Sections were incubated with primary antibody overnight at 4 °C. Next, primary antibody-conjugated sections were incubated with specified secondary antibodies, Alexa Fluor 594 (Abcam, cat# ab150116, 1:300), Alexa Fluor 488 (Thermofisher, cat# A48262, 1:200), or Alexa Fluor 488 (Abcam, cat# ab150077, 1:300), for 1 h at 37 °C in the dark. Negative control was incubated with the secondary antibody without the primary antibody. All sections were stained with DAPI (Thermofisher, cat# P36931) for nucleus counterstain.

High-resolution images of each section were captured and digitized using a sophisticated microscopic imaging system (Leica DM5500; Leica Micro-systems, Wetzlar, Germany).

### IPFP removal (IPFPx)

Twelve-week-old male *C57BL/6* mice were anesthetized for DMM surgery and allowed a two-week recovery period. At the end of this period, the IPFP was excised. The incisions were meticulously closed in layers using 7-0 prolene sutures and 5-0 vicryl sutures in an interrupted pattern. All procedures were conducted under aseptic conditions. The mice were sacrificed at week six following the IPFPx surgery.

### Gait analysis

Gait analysis was conducted using the Catwalk system (Noldus, Wageningen, Netherlands) to evaluate joint function [48]. Prior to the formal experiments, the mice were trained to become familiar with the glass walkway to ensure accurate image capture. During the analysis, paw prints of the left forelimb, right forelimb, left hind limb, and right hind limb were automatically recorded by the system’s built-in software as the mice traversed the region of interest (ROI). A successful recording for each mouse required at least three trials, with each trial consisting of an average of three complete step cycles, free from hesitation or interruption, and allowing for a maximum speed variation of 30%. The parameters collected and analyzed included single stance, stand, maximal contact area, maximal contact AT, swing, maximal intensity AT, duty cycle, and stride length.

### Intra-articular injection of Neu Ab

The skin of 12-week-old male *C57BL/6* mice were treated with betadine following anesthesia with the xylazine (4 mg/kg, i.p.) and ketamine (60 mg/kg, i.p.). To neutralize IL-6 derived from the IPFP, an IL-6-neutralizing antibody (Neu Ab, 2 μg/unilateral/3 days, Thermofisher, cat# AMC0862) was locally injected into the subcapsular IPFP without surgical incision on day 4 post-DMM surgery [10]. As a control, DMM mice received a corresponding injection of IgG (15 μL/unilateral/3 days, Thermofisher, cat# 14-4301-85, dilution for 3.75 folds with saline) [49]. All mice were sacrificed on day 14 post-DMM surgery via intraperitoneal injection of an overdose of sodium pentobarbital.

### Intra-articular injection of IL-6

The skin of 12-week-old male *C57BL/6* mice were treated with betadine following anesthesia with the xylazine (4 mg/kg, i.p.) and ketamine (60 mg/kg, i.p.). Recombinant IL-6 (1 μg/unilateral/4 days, MCE, cat# HY-P7063) was locally injected into the subcapsular IPFP without a surgical incision on day 0 [10, 49]. As a control, mice received a corresponding injection of saline (15 μL/unilateral/4 days). All mice were sacrificed on day 14 via intraperitoneal injection of an overdose of sodium pentobarbital.

### Conditioned medium

DMM or Sham mice were sacrificed to collect the IPFP on day 14 post surgery and injection. All procedures were conducted under aseptic conditions. The IPFP was immersed in 0.6 mL α-Minimum Essential Medium (α-MEM) supplemented with 2% fetal bovine serum (FBS, Gibco, cat# 16140071) and 1% penicillin-streptomycin-neomycin (PSN, Gibco, cat# 15640055), and incubated for 2 days at 37 °C in a 5% CO_2_ atmosphere. The medium was then centrifuged at 600 g for 3 minutes, and the supernatant was stored at -80 °C until further analysis.

### Enzyme-linked immunosorbent assay (ELISA)

The level of OPN in the conditioned medium was quantified using an OPN ELISA kit (Abcam, cat# ab100734). The stored supernatant was diluted 25-fold with the provided diluent buffer. The optical density in each well was immediately measured at 450 nm using a microplate reader (BioTek Quant Microplate Spectrophotometer, USA).

### Intra-articular injection of siRNA *Cd61* or siRNA *Spp1*

The small interfering RNA targeting *spp1* (siRNA *Spp1*, Thermo Fisher Scientific, cat# 4390771, Assay ID: s74321) or *Cd61* (siRNA *Cd61*, Thermo Fisher Scientific, cat# AM16708, Assay ID: 156954) was dissolved in nuclease-free sterile water to prepare a siRNA solution at a concentration of 20 μM [10]. Subsequently, the siRNA solution was diluted 7.5-fold with nuclease-free sterile water and combined with transfection reagent (Thermo Fisher Scientific, cat# 13778-075) following the manufacturer’s instructions. The control group was transfected with negative control siRNA (Thermo Fisher Scientific, cat# AM4611). Starting on day 4, mice received 15 μL of siRNA via a 29G needle into the subcapsular IPFP without a surgical incision [10].

### Isolation of primary chondrocytes

Chondrocytes were isolated from 5-week-old mice [50]. Briefly, the cartilage from distal femora and proximal tibiae were dissected free and cut into as small pieces as possible using a stereo zoom microscope. The cartilage fragments were pre-digested for 30 minutes with 0.1% trypsin (Gibco, USA, cat# 25200056) at 37 °C on a shaker. The fragments were collected by centrifugation at 1000 rpm for 5 min and washed once with PBS. Subsequently, the fragments were digested in a 0.2% collagenase A solution (Roche, cat# 10103578001) in FBS-free α-MEM with 1% PSN for 4 h on a shaker. The digestion was halted by adding α-MEM containing 10% FBS. The cell suspension was washed once with PBS and suspended in α-MEM containing 10% FBS and 1% PSN at 37 °C and 5% CO_2_ atmosphere. Chondrocytes at passage 1 were used for experiments.

### Co-culture IPFP and chondrocytes

Pre-treated IPFP with intra-IPFP injection of siRNA was collected for co-culture [33, 51]. All procedures were performed under sterile conditions. For co-culture of chondrocytes with IPFP, chondrocytes were seeded in the lower chamber of a 12-well plate containing α-MEM supplemented with 10% FBS and 1% PSN, and incubated at 37 °C in a 5% CO_2_ atmosphere. A transwell insert (0.4 μm polycarbonate filter, Corning, cat# CLS3401-48EA) was placed in the upper chamber, where a single IPFP was cultured, sharing the medium with the lower chamber. After 2 days of co-culture, chondrocytes were collected for mRNA expression analysis.

### Total RNA extraction

Using ophthalmic instruments to isolate the IPFP depot under a stereo zoom microscope. IPFPs were pooled from six mice and immersed in 0.5 mL TRIzol solution (Invitrogen, cat# 15596026). IPFPs were homogenized by KIMBLE Dounce tissue grinder (Sigma, USA, cat# D8938) on ice [52, 53]. The precipitation was digested for another 15 min and kept on the ice.

Cartilage samples were meticulously collected from the medial tibial plateau and the lowest point of the medial femoral condyle using ophthalmic instruments under a stereo zoom microscope. The cartilage tissues from four mice were pooled and subsequently immersed in 0.5 mL TRIzol solution. Cartilage was homogenized by KIMBLE Dounce tissue grinder on ice. The precipitation was digested for another 30 min and kept on the ice.

Total RNA was extracted and quantified with a NanoDrop spectrophotometer (Wilmington, DE, USA). Subsequently, 500 ng of RNA was reversely transcribed into cDNA using a cDNA synthesis kit from Takara [10].

### Quantitative RT-PCR

To quantify the mRNA expression, quantitative reverse transcription PCR (qRT-PCR) analysis was conducted using specific primers for mouse genes matched to cDNA on a QuantStudio^TM^ 12 K Flex Real-time PCR system (Life Technologies, Thermo Fisher Scientific) (Supplementary Table 1). The specificity of each qRT-PCR reaction was validated through the analysis of melting curves. Relative gene expression was normalized to that of a positive control and calculated using the 2^−△△Ct^ method.

### Statistical analysis

To estimate the minimum sample size required for both in vitro and in vivo experiments, we performed power calculations based on preliminary data from our pilot study. Allocation and treatment procedures were unblinded; however, all analyses were performed in a blinded fashion by researchers blinded to group assignments. Mice exhibiting a weight deviation exceeding 20% within the same group, poor sample quality, contamination, or equipment failure were excluded from the analysis. GraphPad Prism 8.2.1 software was used for statistical analyses. Data are presented as mean ± standard deviation (SD), and statistical significance was defined as *p* < 0.05. A two-tailed *Student’s t-test* was employed to compare two experimental groups. For multiple group comparisons, one-way ANOVA with *Tukey’s post hoc* test or two-way ANOVA with *Tukey’s post hoc* test was utilized. Additionally, two-way ANOVA followed by *Sidak’s post hoc* test was conducted for repeated measures. Details regarding sample size and replication were provided in the figure legends. All in vitro experiments were repeated a minimum of three times.

## Supplementary information

Supplementary materials

## Data Availability

The datasets generated and/or analyzed during the current study are available from the corresponding author upon reasonable request.
