# Supplementary material for: Fat-cartilage axis: the regulation of IL-6/Osteopontin signaling in osteoarthritis of mice
Source: Cell Death Discov. 2025 Jul 15;11:325. doi: 10.1038/s41420-025-02622-6 (PMC12263885; doi:10.1038/s41420-025-02622-6)
Supplement: Supplementary file 1 — Supplementary materials [file 41420_2025_2622_MOESM1_ESM.docx]

**Supplementary Information**

**Fat-cartilage axis: the regulation of IL-6/Osteopontin signaling in osteoarthritis of mice**

**Dai *et al.***

Table S1. Primer sequences used for qRT-PCR.

**Table S1. Primer sequences used for qRT-PCR**

| Gene name | Oligonucleotide primers |
| --- | --- |
| *Spp1*-F | AGCAAGAAACTCTTCCAAGCAA |
| *Spp1*-R | GTGAGATTCGTCAGATTCATCCG |
| *Sox9*-F | GAGCCGGATCTGAAGAGGGA |
| *Sox9*-R | GCTTGACGTGTGGCTTGTTC |
| *Acan*-F | CCTGCTACTTCATCGACCCC |
| *Acan*-R | AGATGCTGTTGACTCGAACCT |
| *Il-6*-F | TAGTCCTTCCTACCCCAATTTCC |
| *Il-6*-R | TTGGTCCTTAGCCACTCCTTC |
| *Tnfa*-F | CCTGTAGCCCACGTCGTAG |
| *Tnfa*-R | GGGAGTAGACAAGGTACAACCC |
| *Mmp9*-F | CTGGACAGCCAGACACTAAAG |
| *Mmp9*-R | CTCGCGGCAAGTCTTCAGAG |
| *Mmp13*-F | TGTTTGCAGAGCACTACTTGAA |
| *Mmp13*-R | CAGTCACCTCTAAGCCAAAGAAA |
| *Actb*-F | GGCTGTATTCCCCTCCATCG |
| *Actb*-R | CCAGTTGGTAACAATGCCATGT |
| *Runx2*-F | TTCAACGATCTGAGATTTGTGGG |
| *Runx2*-R | GGATGAGGAATGCGCCCTA |
| *Timp1*-F | GCAACTCGGACCTGGTCATAA |
| *Timp1*-R | CGGCCCGTGATGAGAAACT |
| *Col1a1*-F | GCTCCTCTTAGGGGCCACT |
| *Col1a1*-R | CCACGTCTCACCATTGGGG |
| *Col1a2*-F | GTAACTTCGTGCCTAGCAACA |
| *Col1a2*-R | CCTTTGTCAGAATACTGAGCAGC |
| *Col2a1*-F | GGGAATGTCCTCTGCGATGAC |
| *Col2a1*-R | GAAGGGGATCTCGGGGTTG |
| *Col3a1*-F | CTGTAACATGGAAACTGGGGAAA |
| *Col3a1*-R | CCATAGCTGAACTGAAAACCACC |
| *Col6a1*-F | CTGCTGCTACAAGCCTGCT |
| *Col6a1*-R | CCCCATAAGGTTTCAGCCTCA |
| *Col10a1*-F | TTCTGCTGCTAATGTTCTTGACC |
| *Col10a1*-R | GGGATGAAGTATTGTGTCTTGGG |
| *Adamts5*-F | GGAGCGAGGCCATTTACAAC |
| *Adamts5*-R | CGTAGACAAGGTAGCCCACTTT |
| *Tgfb1*-F | CTCCCGTGGCTTCTAGTGC |
| *Tgfb1*-R | GCCTTAGTTTGGACAGGATCTG |
| *Tgfb2*-F | CTTCGACGTGACAGACGCT |
| *Tgfb2*-R | GCAGGGGCAGTGTAAACTTATT |
| *Cd61*-F | CCACACGAGGCGTGAACTC |
| *Cd61*-R | CTTCAGGTTACATCGGGGTGA |
| *Cd51*-F | CCGTGGACTTCTTCGAGCC |
| *Cd51*-R | CTGTTGAATCAAACTCAATGGGC |
